# Evaluation of Crocetin as a Protective Agent in High Altitude Hypoxia-Induced Organ Damage

**DOI:** 10.3390/ph17080985

**Published:** 2024-07-25

**Authors:** Jun Yang, Kai Luo, Ziliang Guo, Renjie Wang, Qingyuan Qian, Shuhe Ma, Maoxing Li, Yue Gao

**Affiliations:** 1 College of Pharmacy, Gansu University of Chinese Medicine, Lanzhou 730000, China; yangjunzy@163.com (J.Y.); 18294623280@163.com (K.L.); wangrenjiegy@163.com (R.W.); mclsxka@163.com (S.M.); 2 Department of Pharmaceutical Sciences, Beijing Institute of Radiation Medicine, Beijing 100850, China; q15888906749@163.com; 3 College of Pharmacy, Lanzhou University, Lanzhou 730000, China; guozl20@lzu.edu.cn; 4 National Key Laboratory of Kidney Diseases, Beijing 100850, China

**Keywords:** crocetin, anti-hypoxia, antioxidant, anti-inflammatory

## Abstract

Crocetin is an aglycone of crocin naturally occurring in saffron and has been proved to have antioxidant, anti-inflammatory, and antibacterial activities. In this experiment, the protective effect of crocetin on vital organs in high-altitude hypoxia rats was studied. Crocetin was prepared from gardenia by the alkaline hydrolysis method, and its reducing ability and free radical scavenging ability were tested. The in vitro anti-hypoxia vitality was studied on PC_12_ cells. The anti-hypoxic survival time of mice was determined in several models. The acute hypoxic injury rat model was established by simulating the hypoxic environment of 8000 m-high altitude for 24 h, and the anti-hypoxia effect of crocetin was evaluated by intraperitoneal injection with the doses of 10, 20, and 40 mg/kg. The water contents of the brain and lung were determined, and the pathological sections in the brain, lung, heart, liver, and kidney were observed by HE staining. The levels of oxidative stress (SOD, CAT, H_2_O_2_, GSH, GSH-Px, MDA) and inflammatory factors (IL-1β, IL-6, TNF-α, VEGF) in rat brain, lung, heart, liver, and kidney tissues were detected by ELISA. The results indicated that crocetin exhibited strong reducing ability and free radical scavenging ability and could improve the activity of PC_12_ cells under hypoxia. After intraperitoneal injection with crocetin, the survival time of mice was prolonged, and the pathological damage, oxidative stress, and inflammation in rats’ tissue were ameliorated. The protective activity of crocetin on vital organs in high-altitude hypoxia rats may be related to reducing oxidative stress and inhibiting inflammatory response.

## 1. Introduction

High-altitude regions attract millions of people each year for recreational reasons, such as sightseeing, trekking, climbing, and skiing, or even to escape heat waves. In 2014 alone, >15 million tourists travelled to Lhasa, Tibet, which is at 3658 m above sea level. In addition, many thousands of workers, soldiers, and pilgrims are temporarily exposed to high altitudes, and >80 million people live permanently in places above 2500 m [1]. The air pressure decreases exponentially with the increase of altitude. The exposure to the plateau environment will lead to hypoxia in tissues, organs, and bodies, which will lead to energy metabolism disorders, mitochondrial damage, cell apoptosis, oxidative stress, and inflammation outbreak. The main physiological responses of the body’s acute hypoxia exposure include hyperventilation [2] triggered by the hypoxic ventilatory response, which causes sympathetic activation with an increase in heart rate and cardiac output [3], pulmonary vasoconstriction, and elevated pulmonary artery pressure [4]. Overloaded and long-term physiological responses may contribute to the occurrence of high-altitude diseases, including acute mountain sickness (AMS), high-altitude hypertension (HAH), high-altitude heart disease (HAHD), high-altitude cerebral edema (HACE), and high-altitude pulmonary edema (HAPE) [5].

Plenty of studies have proved that acute or chronic high-altitude hypoxia may induce the decrease of the activity of antioxidant capacity biomarkers, such as superoxide dismutase (SOD), catalase (CAT), glutathione peroxidase/reductase (GSH-Px), peroxiredoxin/thioredoxin (PRX/Trx), and metalloproteases (MMPs), and then lead to the increase of oxidative stress biomarkers, such as malondialdehyde (MDA) and reactive oxygen species (ROS) [6,7,8,9]. Oxidative stress and inflammation are mutual influences. Hypoxia causes oxidative stress, which in turn leads to the production of many inflammatory cytokines such as cytokine interleukin-6 (IL-6), cytokine interleukin-1β (IL-1β), tumor necrosis factor-α (TNF-α), vascular endothelial cell growth factor (VEGF), hypoxia inducible factor-1 α (HIF-1α), etc. [10,11]. It should be emphasized that systemic oxidative stress and inflammatory outbreaks caused by high-altitude hypoxia are important factors leading to damage of important tissues and organs, which manifests as various diseases related to high-altitude exposure.

Saffron (*Crocus sativus* L.) is a valuable traditional medicine of Persia and China, which has been used for thousands of years. Crocin and its aglycone crocetin are characteristic pigment components in saffron, which have been proved with cardioprotective, hepatoprotective, neuroprotective, antinociceptive, antidepressant, antiviral, anticancer, atherosclerotic, antidiabetic, and memory enhancing properties [12].

Miraculously, the fruit of traditional Chinese medicine *Gardenia jasminoides* J. Ellis also contains a large amount of crocin [12]. In the past decade, our group established enrichment and purification technology for the first time to obtain gardenia yellow pigment with high content of crocin. In vitro and in vivo experiments proved this gardenia yellow pigment had strong radical scavenging ability, antioxidant activity, and anti-hypoxia and anti-fatigue effects with mechanisms to reduce the accumulation of bad metabolites, increase energy reserves, improve free radical scavenging capacity, increase related metabolic enzyme activities, inhibit the infiltration of inflammatory cells, and reduce apoptosis under simulated high-altitude hypoxic environments [13]. Preliminary studies of our group have found that after 400 mg/kg administration of crocin-I to normoxic and hypoxic rats, not only crocin-I, but also crocetin can be detected with considerable content in the liver, lung, spleen, testis, kidney, heart, and brain tissue. Crocin-I may be absorbed and hydrolyzed by intestinal flora and liver into crocetin [14].

Based on our group’s study of crocin-I’s metabolism in rats under normoxic and hypoxic conditions, we propose that crocetin may be the ultimate anti-hypoxia material basis of crocin-I, gardenia yellow pigment, and saffron. This paper investigates the anti-hypoxia effects of crocetin through various in vivo and in vitro experiments and examines its protective effects on vital organs in rats subjected to simulated high-altitude hypoxia, thereby providing a theoretical foundation for the use of crocetin in combating high-altitude hypoxia.

## 2. Results

### 2.1. Reducing Ability and Radical Scavenging Ability

**Figure 1:**
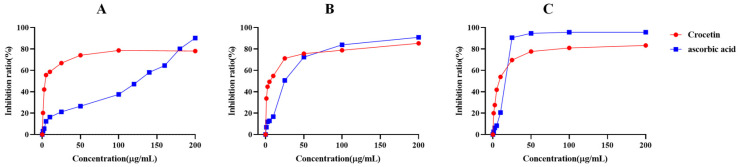
Reducing ability and radical scavenging ability of crocetin. (**A**) Inhibition of •O_2_^−^, (**B**) inhibition of H_2_O_2_, (**C**) inhibition of •OH^−^.

As shown in Figure 1A, the inhibitory effect of crocetin on •O_2_^−^ increased, and the effect of the system was much higher than that of ascorbic acid. Before 180 μg/mL, the luminescence inhibitory effect of crocetin on the system was much greater than the ascorbic acid.

As shown in Figure 1B, the inhibition rate of crocetin increased rapidly with increasing concentration. In the range of 1.00 to 50.00 μg/mL, the inhibition rate of crocetin on H_2_O_2_ was greater than ascorbic acid, and the inhibition rate of ascorbic acid over 50.00 μg/mL was greater than that of crocetin after 50.00 μg/mL.

The experiments found that the IC_50_ of crocetin on •O_2_^−^, H_2_O_2_, and •OH^−^ were 6.39, 4.67, and 8.63 μg/mL, respectively (Table 1). The results showed that crocetin had strong antioxidant activities, and compared with the control group ascorbic acid, there was a very significant difference (*p* < 0.001).

### 2.2. Effect of Crocetin on PC_12_ Cell Viability

**Figure 2:**
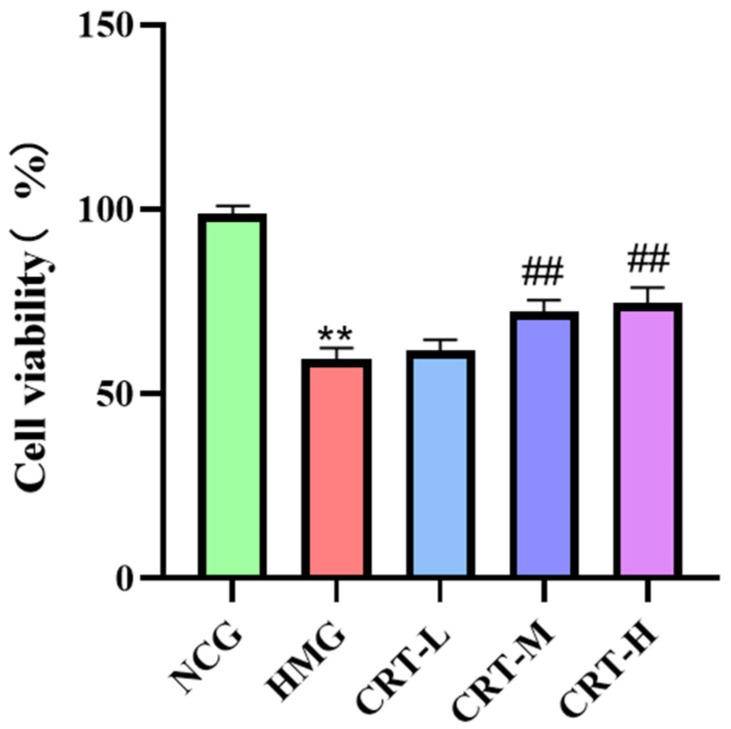
Effect of crocetin on hypoxic injury activity of PC_12_ cell. Data are expressed as mean ± SD (*n* = 3/group). Note: Compared to the NC, ** *p* < 0.01; compared with HM, ^##^ *p* < 0.01. NCG: normoxic control group; HMG: hypoxia model group; CRT-L: crocetin low-dose group (25 μmol/L); CRT-M: crocetin medium-dose group (50 μmol/L); CRT-H: crocetin high-dose group (100 μmol/L).

As shown in Figure 2, compared with the normoxic control group, the viability of PC_12_ cells decreased significantly in the hypoxia model group (*p* < 0.01). After administration with crocetin, both the CRT-M group and the CRT-H group could significantly increase PC_12_ cell viability (*p* < 0.01). The results showed that crocetin could improve the viability of PC_12_ cells under hypoxia.

### 2.3. Effect of Crocetin on Hypoxic Mice

**Figure 3:**
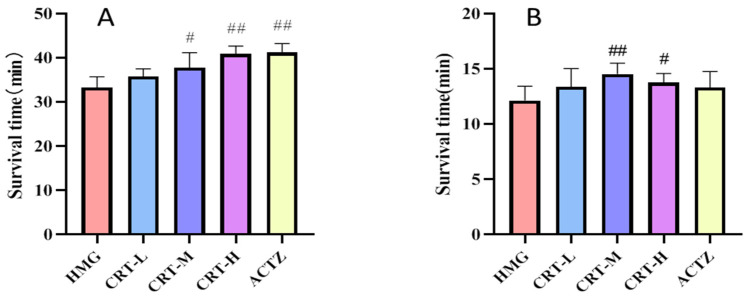
Effect on the survival time of hypoxia mice by intraperitoneal injection crocetin. Data are expressed as mean ± SD (*n* = 10/group). Note: compared with HM, ^#^ *p* < 0.05, ^##^ *p* < 0.01. HMG: hypoxia model group; CRT-L: crocetin low-dose group; CRT-M: crocetin medium-dose group; CRT-H: crocetin high-dose group; ACTZ: positive control group. (**A**) The close normobaric hypoxia experiment. (**B**) The sodium nitrite hypoxia experiment.

As shown in Figure 3A, the result of normobaric hypoxia test indicate that low, medium, and high CRT doses can effectively prolong the survival time of mice in a dose-dependent manner; prolonged rates were 5.56%, 13.57% (*p* < 0.05), and 22.90% (*p* < 0.01), respectively.

The results of the sodium nitrite hypoxia experiment are shown in Figure 3B. Compared with the HM group, the prolongation rates of CRT-L, CRT-M, and CRT-H groups are 10.30%, 19.88% (*p* < 0.01), and 13.52%, respectively. The prolongation rate of the ACTZ group is 9.58%. Among them, the CRT-M group and CRT-H group (*p* < 0.05) can significantly prolong the time to live of hypoxic mice, indicating that crocetin can significantly improve the hypoxia tolerance of mice and has certain anti-hypoxia activity.

### 2.4. Protective Effect of Crocetin on High Altitude Hypoxia Rats

#### 2.4.1. Observation on Survival State of Hypoxia Rats

During the whole process of hypoxia injury, the respiration amplitude of rats decreased, and the respiration frequency increased significantly. Rats in the HMG manifested significant changes, including less food and water intake, hair disorder, slow movement, and the death of two rats.

Except for one rat that died in the CRT-L group, the rats in other crocetin administration groups and ACTZ had relatively less food and water intake, better activity state, slightly disordered hair, and rapid breathing. The rats in the NCG had normal feeding, water intake, and other activities.

#### 2.4.2. Effect of Crocetin on Brain and Lung Water Content in Hypoxic Rats

**Figure 4:**
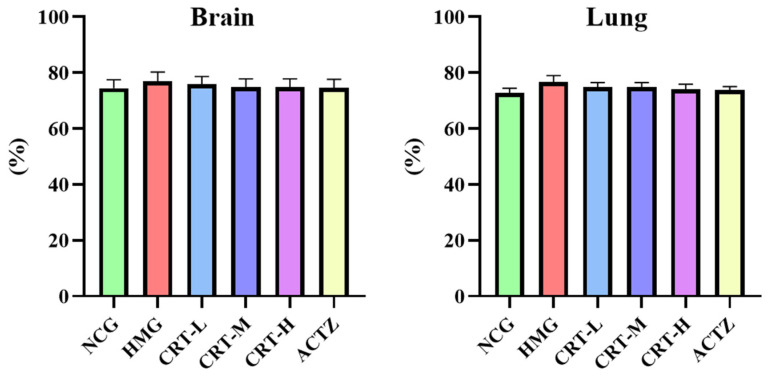
The effect of crocetin on brain and lung water content in high-altitude hypoxia rats. Data are expressed as mean ± SD (*n* = 10/group). Note: NCG: normal oxygen control group; HMG: hypoxia model group; CRT-L: crocetin low-dose group; CRT-M: crocetin medium-dose group; CRT-H: crocetin high-dose group; ACTZ: positive control group.

The results of crocetin anti-hypoxia activity are shown in Figure 4; compared with NCG, the brain water content of rats with HMG increased by 3.32%, and the lung water content increased by 5.23%, but the difference was not significant (*p* > 0.05). Compared with HMG, the brain and lung water contents in crocetin treatment groups and the acetazolamide group had a certain downward trend; the brain water content decreased by 1.32%, 2.74%, 2.78%, and 3.13% in the CRT-L, CRT-M, CRT-H, and ACTZ groups, respectively. The lung water content decreased by 2.26%, 2.32%, 3.22%, and 3.85% in the CRT-L, CRT-M, CRT-H, and ACTZ group groups, respectively, but there was no significant difference between the above groups (*p* > 0.05).

#### 2.4.3. Effect of Crocetin on Pathological Structure of Tissues in Hypoxic Rats

**Figure 5:**
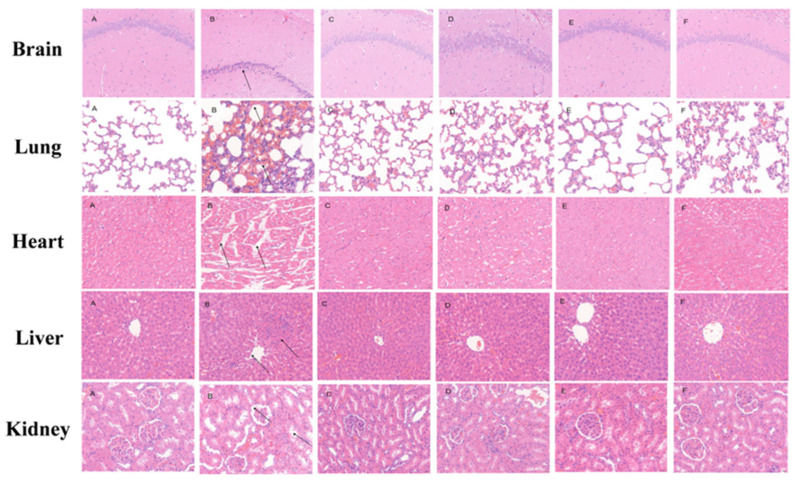
Histopathological changes of vital organs in high-altitude hypoxia rats (×400). (**A**) Normoxic control group (NCG), (**B**) hypoxia model group (HMG), (**C**) crocetin low-dose group (CRT-L, 10 mg/kg), (**D**) crocetin medium-dose group (CRT-M, 20 mg/kg), (**E**) crocetin high-dose group (CRT-H, 40 mg/kg), (**F**) acetazolamide group (ACTZ, 70 mg/kg).

The histopathological effects of crocetin on simulated high-altitude hypoxia rats are shown in Figure 5, compared with NCG, the arrangement of hippocampus cells was disordered, the number of hippocampus cells decreased, the gap of nuclear-cytoplasmic increased, and the nucleus showed pyknosis into an irregular shape in HMG. After CRT and ACTZ drug intervention, the pathological structure of brain tissue was improved to a certain extent; the cell structure tended to be normal, the number of neurons increased, and the nuclear staining decreased.

The structure of lung tissue was regular, the alveolar cavity was clear, and there was no obvious edema, inflammation, or other pathological damage in NCG. Compared with NCG, the lung tissue of HMG rats was significantly damaged, the structural integrity was destroyed, the alveolar wall was obviously thickened, and the pulmonary interstitium was seriously congested. Correspondingly, the pathological structure of lung tissue was improved, the alveolar wall was slightly thickened, the alveolar cavity was dilated and tended to be normal, and the inflammatory reaction was reduced in the drug intervention group.

The myocardial cells of NCG group rats have clear boundaries, visible myofibrils and striations, and clear nuclei. Compared with NCG, the heart tissue of HMG rats was accompanied by a small amount of inflammatory cell infiltration. Correspondingly, the pathological structure of the heart tissue in the drug intervention group was alleviated, and myocardial cells tended to be normal without significant inflammatory cell infiltration.

The hepatocytes of NCG were arranged in a radial and compact manner with the central vein as the center, the morphology and structure of cells were clear and complete without pathological damage. The hepatocytes of HMG were swollen and loosely arranged, the structure of hepatic lobules was abnormal, central veins were disordered, inflammatory cells infiltrated in the portal area, and fibrous tissue proliferated. After drug intervention, the pathological structure of rat liver tissue was improved. The hepatocytes were arranged in an orderly fashion and tended to be normal, accompanied by mild granular degeneration.

The kidney tissue cells in NCG were clear, the structure was neat, and the shape of glomerulus was regular. Meanwhile, there was obvious glomerular basement membrane hyperplasia and interstitial swelling in HMG. Compared with HMG, the structure of the administration group was orderly arranged, the cell swelling was reduced, and the proliferation of the glomerular basement membrane was alleviated.

#### 2.4.4. Inhibition of Crocetin on Tissues Peroxidation Damage in Hypoxic Rats

##### Effect of Crocetin on Oxidative Stress Markers in Brain Tissue of Hypoxic Rats

**Figure 6:**
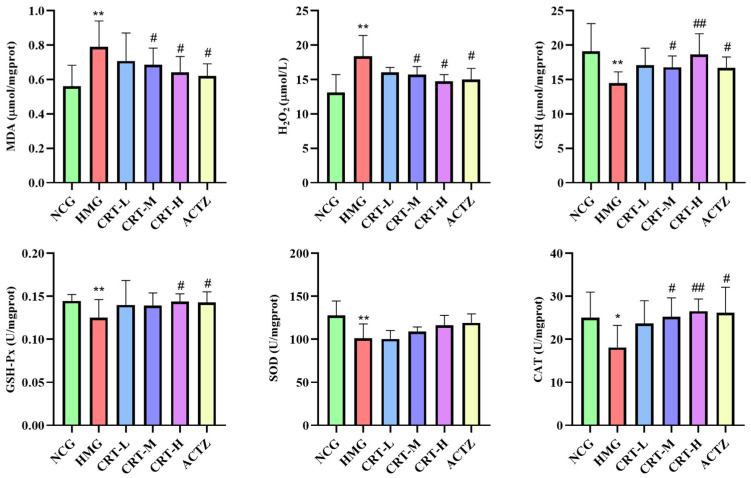
Crocetin regulated the oxidative stress index in hypoxic-injured brain tissue. Data are expressed as mean ± SD (*n* = 8/group). Note: * *p* < 0.05 NCG vs. HMG; ** *p* < 0.01 NCG vs. HMG; ^##^ *p* < 0.01, ^#^ *p* < 0.05, HMG vs. medication groups. NCG: normal oxygen control group; HMG: hypoxia model group; CRT-L: crocetin low-dose group; CRT-M: crocetin medium-dose group; CRT-H: crocetin high-dose group; ACTZ: positive control group.

As shown in Figure 6, compared with NCG, the content of MDA and H_2_O_2_ in the brains of HMG rats was significantly increased, reaching 41.10% and 40.45% (*p* < 0.01), respectively. The content of GSH (*p* < 0.01) and the activity of GSH-Px (*p* < 0.01), SOD (*p* < 0.01), CAT (*p* < 0.05) in the brain of HMG rats was significantly decreased by 24.27%, 13.42%, 20.55%, and 27.61%, respectively.

After giving crocetin intervention, compared with HMG, the content of MDA in CRT-M, CRT-H, and ACTZ groups decreased by 13.10%, 18.82%, and 21.42%, respectively. There were significant differences (*p* < 0.05). The content of H_2_O_2_ in CRT-M, CRT-H, and ACTZ groups decreased by 14.56%, 19.69%, 18.40%, respectively. There were significant differences (*p* < 0.05). The content of GSH in CRT-M, CRT-H, and ACTZ groups increased by 16.02%, 28.83%, and 15.43%, respectively. There were significant differences (*p* < 0.05 or *p* < 0.01). The activity of GSH-Px in CRT-H and ACTZ groups increased by 14.63% and 14.01%, respectively. There were significant differences (*p* < 0.05). The activity of SOD in CRT-L, CRT-M, CRT-H, and ACTZ groups increased by 1.09%, 7.58%, 14.49%, and 17.47%, respectively. However, there was no significant difference (*p* > 0.05). The activity of CAT in CRT-M, CRT-H, and ACTZ groups increased by 39.19%, 46.47%, and 44.62%, respectively. There were significant differences (*p* < 0.05 or *p* < 0.01).

##### Effect of Crocetin on Oxidative Stress Markers in Lung Tissue of Hypoxic Rats

**Figure 7:**
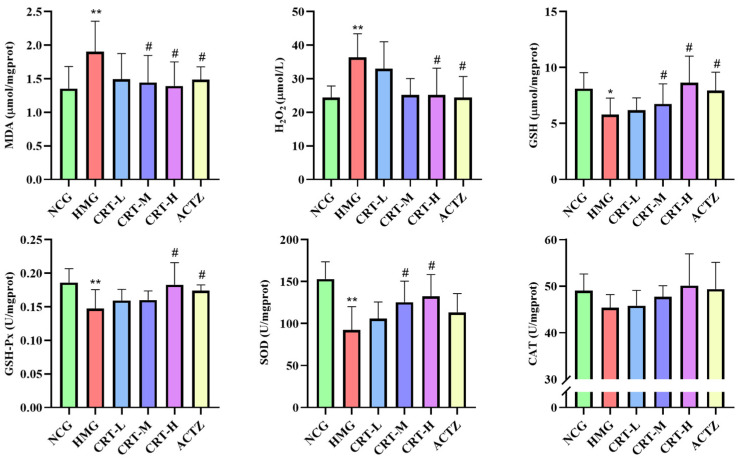
Crocetin regulated the oxidative stress index in hypoxic-injured lung tissue. Data are expressed as mean ± SD (*n* = 8/group). Note: * *p* < 0.05 NCG vs. HMG; ** *p* < 0.01 NCG vs. HMG; ^#^ *p* < 0.05, HMG vs. medication groups. NCG: normal oxygen control group; HMG: hypoxia model group; CRT-L: crocetin low-dose group; CRT-M: crocetin medium-dose group; CRT-H: crocetin high-dose group; ACTZ: positive control group.

As shown in Figure 7, compared with NCG, the content of MDA and H_2_O_2_ in the lungs of HMG rats was significantly increased, reaching 40.73% and 49.39% (*p* < 0.01), respectively. The content of GSH (*p* < 0.05) and the activity of GSH-Px (*p* < 0.01), SOD (*p* < 0.01), and CAT (*p* > 0.05) in the brain of HMG rats was significantly decreased by 28.64%, 20.82%, 39.65%, and 7.53%, respectively.

After giving crocetin intervention, compared with HMG, the content of MDA in CRT-M, CRT-H, and ACTZ groups decreased by 24.16%, 26.79%, and 21.96%, respectively. There were significant differences (*p* < 0.05). The content of H_2_O_2_ in CRT-H and ACTZ groups decreased by 30.71% and 32.82%, respectively. There were significant differences (*p* <0.05). The content of GSH in CRT-M, CRT-H, and ACTZ groups increased by 16.69%, 49.33%, and 37.39%, respectively. There were significant differences (*p* < 0.05). The activity of GSH-Px in CRT-H and ACTZ groups increased by 24.00% and 16.98%, respectively, and there were significant differences (*p* < 0.05).The activity of SOD in CRT-M and CRT-H groups increased by 35.69% and 43.54%, respectively. There were significant differences (*p* < 0.05). The activity of CAT in rat lung tissue increased; however, none of them were significant (*p* > 0.05).

##### Effect of Crocetin on Oxidative Stress Markers in Heart Tissue of Hypoxic Rats

**Figure 8:**
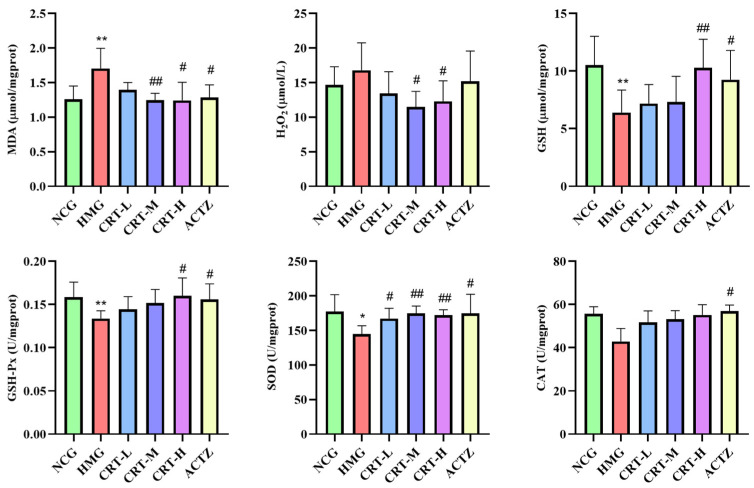
Crocetin regulated the oxidative stress index in hypoxic-injured heart tissue. Data are expressed as mean ± SD (*n* = 8/group). Note: * *p* < 0.05 NCG vs. HMG; ** *p* < 0.01 NCG vs. HMG; ^##^ *p* < 0.01, ^#^ *p* < 0.05, HMG vs. medication groups. NCG: normal oxygen control group; HMG: hypoxia model group; CRT-L: crocetin low-dose group; CRT-M: crocetin medium-dose group; CRT-H: crocetin high-dose group; ACTZ: positive control group.

As shown in Figure 8, compared with NCG, the content of MDA and H_2_O_2_ in the hearts of HMG rats was significantly increased, reaching 35.63% and 14.35% (*p* < 0.01 or *p* < 0.05), respectively. The content of GSH (*p* < 0.01) and the activity of GSH-Px (*p* < 0.01), SOD (*p* < 0.05), and CAT (*p* < 0.05) in the brain of HMG rats was significantly decreased by 39.21%, 15.74%, 18.26%, and 22.91%, respectively.

After giving crocetin intervention, compared with HMG, the content of MDA in CRT-M, CRT-H, and ACTZ groups decreased by 26.86%, 27.13%, and 24.75%, respectively. There were significant differences (*p* < 0.05 or *p* < 0.01). The content of H_2_O_2_ in CRT-M and CRT-H groups decreased by 31.57% and 26.68%, respectively. There were significant differences (*p* < 0.05). The content of GSH in CRT-H and ACTZ groups increased by 60.68% and 44.62%, respectively. There were significant differences (*p* < 0.05 or *p* < 0.01). The activity of GSH-Px in CRT-H and ACTZ groups increased by 19.58% and 16.60%, respectively. There were significant differences (*p* < 0.05). The activity of SOD in CRT-L, CRT-M, CRT-H, and ACTZ groups increased by 15.43%, 20.85%, 18.86%, and 20.78, respectively. There were significant differences (*p* < 0.05 or *p* < 0.01). The activity of CAT in ACTZ groups increased by 32.75%, respectively. There were significant differences (*p* < 0.05).

##### Effect of Crocetin on Oxidative Stress Markers in Liver Tissue of Hypoxic Rats

**Figure 9:**
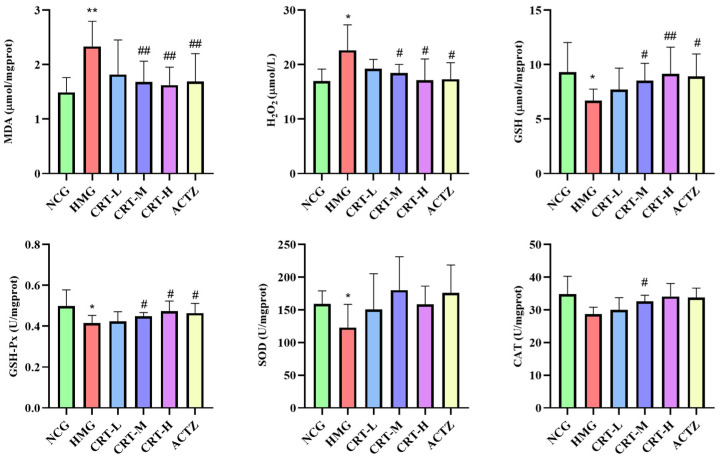
Crocetin regulated the oxidative stress index in hypoxic-injured liver tissue. Data are expressed as mean ± SD (*n* = 8/group). Note: * *p* < 0.05 NCG vs. HMG; ** *p* < 0.01 NCG vs. HMG; ^##^ *p* < 0.01,^#^ *p* < 0.05, HMG vs. medication groups. NCG: normal oxygen control group; HMG: hypoxia model group; CRT-L: crocetin low-dose group; CRT-M: crocetin medium-dose group; CRT-H: crocetin high-dose group; ACTZ: positive control group.

As shown in Figure 9, compared with NCG, the content of MDA and H_2_O_2_ in the livers of HMG rats was significantly increased, reaching 150.52% and 33.29% (*p* < 0.01 or *p* < 0.05), respectively. The content of GSH and the activity of GSH-Px, SOD, and CAT in the brains of HMG rats was significantly decreased by 28.01%, 16.78%, 22.74%, and 17.61% (*p* < 0.05), respectively.

After giving crocetin intervention, compared with HMG, the content of MDA in CRT-M, CRT-H, and ACTZ groups decreased by 37.75%, 50.46%, and 48.92%, respectively. There were significant differences (*p* < 0.01). The content of H_2_O_2_ in CRT-M, CRT-H, and ACTZ groups decreased by 18.61%, 24.33%, and 23.44%, respectively. There were significant differences (*p* < 0.05). The content of GSH in CRT-M, CRT-H, and ACTZ groups increased by 27.10%, 36.69%, and 33.32%, respectively. There were significant differences (*p* < 0.05). The activity of GSH-Px in CRT-M, CRT-H, and ACTZ groups increased by 8.03%, 14.08%, and 11.88%, respectively. There were significant differences (*p* < 0.05). The activity of SOD in CRT-L, CRT-M, CRT-H, and ACTZ groups increased by 22.28%, 46.48%, 28.63%, and 43.21%, respectively. However, there was no significant difference (*p* > 0.05). The activity of CAT in CRT-M groups increased by 13.66% (*p* < 0.05).

##### Effect of Crocetin on Oxidative Stress Markers in Kidney Tissue of Hypoxic Rats

**Figure 10:**
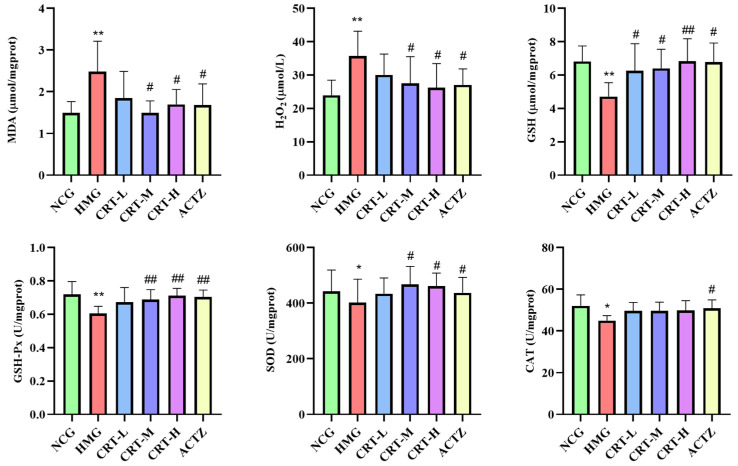
Crocetin regulated the oxidative stress index in hypoxic-injured kidney tissue. Data are expressed as mean ± SD (*n* = 8/group). Note: * *p* < 0.05 NCG vs. HMG; ** *p* < 0.01 NCG vs. HMG; ^##^ *p* < 0.01, ^#^ *p* < 0.05, HMG vs. medication groups. NCG: Normal oxygen control group; HMG: hypoxia model group; CRT-L: crocetin low-dose group; CRT-M: crocetin medium-dose group; CRT-H: crocetin high-dose group; ACTZ: positive control group.

As shown in Figure 10, compared with NCG, the content of MDA and H_2_O_2_ in the kidneys of HMG rats was significantly increased, reaching 56.64% and 49.38% (*p* < 0.01), respectively. The content of GSH (*p* < 0.01) and the activity of GSH-Px (*p* < 0.01), SOD (*p* < 0.05), and CAT (*p* > 0.05) in the brains of HMG rats was significantly decreased by 30.96%, 16.02%, 9.17%, and 13.58% (*p* < 0.05), respectively.

After giving crocetin intervention, compared with HMG, the content of MDA in CRT-M, CRT-H, and ACTZ groups decreased by 28.01%, 30.41%, and 27.51%, respectively. There were significant differences (*p* < 0.01). The content of H_2_O_2_ in CRT-M, CRT-H, and ACTZ groups decreased by 23.23%, 26.87%, and 24.37%, respectively. There were significant differences (*p* < 0.05). The content of GSH in CRT-M, CRT-H, and ACTZ groups increased by 35.97%, 45.01%, and 44.16%, respectively. There were significant differences (*p* < 0.05 or *p* < 0.01). The activity of GSH-Px in CRT-M, CRT-H, and ACTZ groups increased by 13.79%, 17.86%, and 16.46%, respectively. There were significant differences (*p* < 0.01). The activity of SOD in CRT-L, CRT-M, CRT-M, CRT-H, and ACTZ groups increased by 8.15%, 16.32%, 14.89%, and 8.95, respectively. However, there was no significant difference (*p* > 0.05). The activity of CAT in CRT-M groups increased by 10.52% (*p* < 0.05).

#### 2.4.5. Anti-Inflammatory Effect of Crocetin on Tissue of Hypoxic Rats

##### Effect of Crocetin on Inflammatory Factors in Brain Tissue of Hypoxic Rats

**Figure 11:**
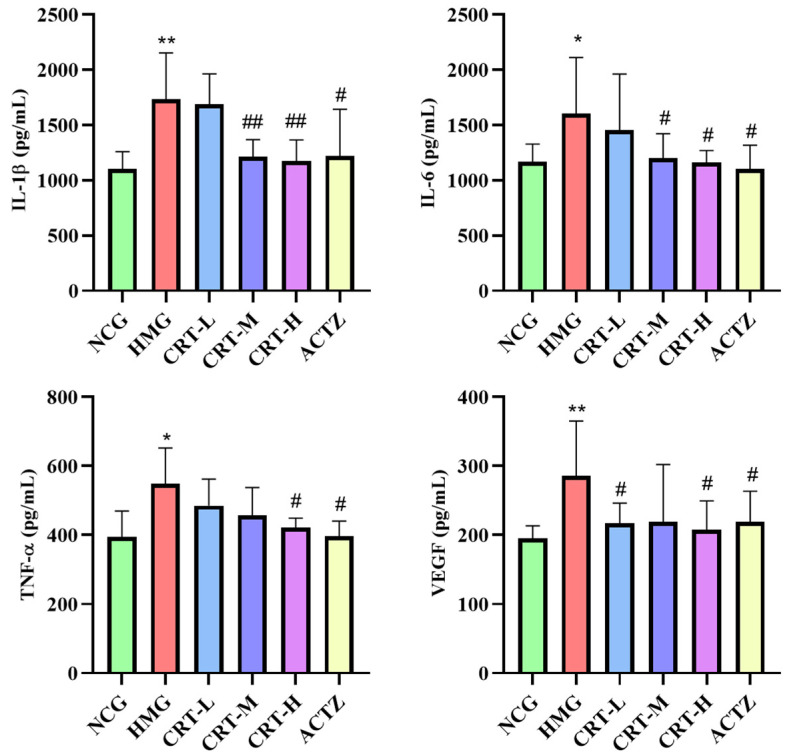
Crocetin reduces inflammatory factor in acute hypoxic injury in rats’ brain tissues. Data are expressed as mean ± SD (*n* = 8/group) Note: ** *p* < 0.01, * *p* < 0.05, NCG vs. HMG; ^##^ *p* < 0.01, ^#^ *p* < 0.05, HMG vs. medication groups; NCG: normal oxygen control group; HMG: hypoxia model group; CRT-L: crocetin low-dose group; CRT-M: crocetin medium-dose group; CRT-H: crocetin high-dose group; ACTZ: positive control group.

As shown in Figure 11, compared with NCG, the content of IL-1β (*p* < 0.01), IL-6 (*p* < 0.05), TNF-α (*p* < 0.05), and VEGF (*p* < 0.01) in the brains of HMG rats was significantly increased by 56.96%%, 37.44%, 38.64%, and 46.12%, respectively.

After drug intervention, compared with HMG, the content of IL-1β in CRT-M, CRT-H, and ACTZ groups decreased by 30.27%, 32.22%, and 29.67%, respectively. There were significant differences (*p* < 0.05 or *p* < 0.01). The content of IL-6 in CRT-M, CRT-H, and ACTZ groups decreased by 25.27%, 27.44%, and 31.17%, respectively. There were significant differences (*p* < 0.05). The content of TNF-α in CRT-H and ACTZ groups decreased by 23.23% and 27.69%, respectively. There were significant differences (*p* < 0.05). The content of VEGF in CRT-L, CRT-H, and ACTZ groups decreased by 24.01%, 27.19%, and 23.28%, respectively. There were significant differences (*p* < 0.05).

##### Effect of Crocetin on Inflammatory Factors in Lung Tissue of Hypoxic Rats

**Figure 12:**
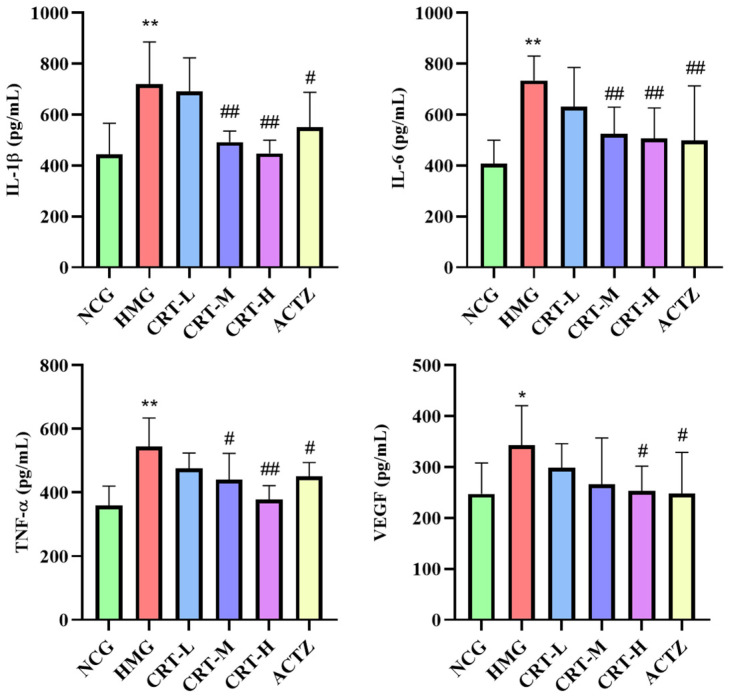
Crocetin reduces inflammatory factor in acute hypoxic injury in rats’ lung tissues. Data are expressed as mean ± SD (*n* = 8/group). Note: ** *p* < 0.01, * *p* < 0.05, NCG vs. HMG; ^##^ *p* < 0.01, ^#^ *p* < 0.05, HMG vs. medication groups; NCG: normal oxygen control group; HMG: hypoxia model group; CRT-L: crocetin low-dose group; CRT-M: crocetin-medium dose group; CRT-H: crocetin high-dose group; ACTZ: positive control group.

As shown in Figure 12, compared with NCG, the content of IL-1β (*p* < 0.01), IL-6 (*p* < 0.01), TNF-α (*p* < 0.01), and VEGF (*p* < 0.05) in the lung of HMG rats was significantly increased by 56.96%, 79.98%, 51.71%, and 38.84%, respectively.

After drug intervention, compared with HMG, the content of IL-1β in CRT-M, CRT-H, and ACTZ groups decreased by 30.27%, 32.22%, and 29.67%, respectively. There were significant differences (*p* < 0.05 or *p* < 0.01). The content of IL-6 in CRT-M, CRT-H, and ACTZ groups decreased by 28.57%, 31.15%, and 32.02%, respectively. There were significant differences (*p* < 0.01). The content of TNF-α in CRT-M, CRT-H, and ACTZ groups decreased by 19.13%, 30.65%, and 17.42%, respectively. There were significant differences (*p* < 0.05 or *p* < 0.01). The content of VEGF in CRT-H and ACTZ groups decreased by 26.40% and 27.69%, respectively. There were significant differences (*p* < 0.05).

##### Effect of Crocetin on Inflammatory Factors in Heart Tissue of Hypoxic Rats

**Figure 13:**
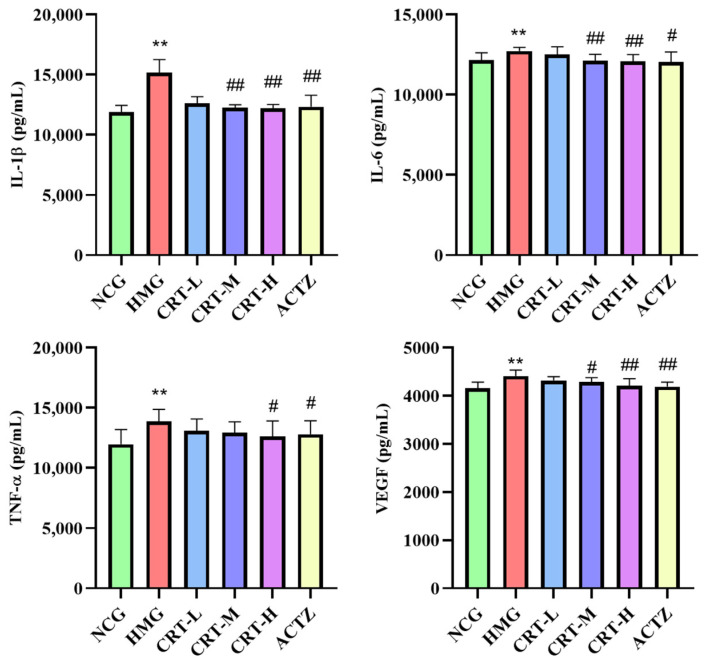
Crocetin reduces inflammatory factor in acute hypoxic injury in rats’ heart tissues. Data are expressed as mean ± SD (*n* = 8/group) Note: ** *p* < 0.01, NCG vs. HMG; ^##^ *p* < 0.01, ^#^ *p* < 0.05, HMG vs. medication groups; NCG: normal oxygen control group; HMG: hypoxia model group; CRT-L: crocetin low-dose group; CRT-M: crocetin medium-dose group; CRT-H: crocetin high-dose group; ACTZ: positive control group.

As shown in Figure 13, Compared with NCG, the content of IL-1β (*p* < 0.01), IL-6 (*p* < 0.01), TNF-α (*p* < 0.01), and VEGF (*p* < 0.01) in the hearts of HMG rats was significantly increased by 27.46%, 45.83%, 15.93%, and 6.19%, respectively.

After drug intervention, compared with HMG, the content of IL-1β in CRT-M, CRT-H, and ACTZ groups decreased by 19.21%, 19.58%, and 18.59%, respectively. There were significant differences (*p* < 0.01). The content of IL-6 in CRT-M, CRT-H, and ACTZ groups decreased by 4.68%, 5.04%, and 5.30%, respectively. There were significant differences (*p* < 0.05 or *p* < 0.01). The content of TNF-α in CRT-H and ACTZ groups decreased by 9.16% and 7.79%, respectively. There were significant differences (*p* < 0.05). The content of VEGF in CRT-M, CRT-H, and ACTZ groups decreased by 2.75%, 4.66%, and 5.04%, respectively. There were significant differences (*p* < 0.05 or *p* < 0.01).

##### Effect of Crocetin on Inflammatory Factors in Liver Tissue of Hypoxic Rats

**Figure 14:**
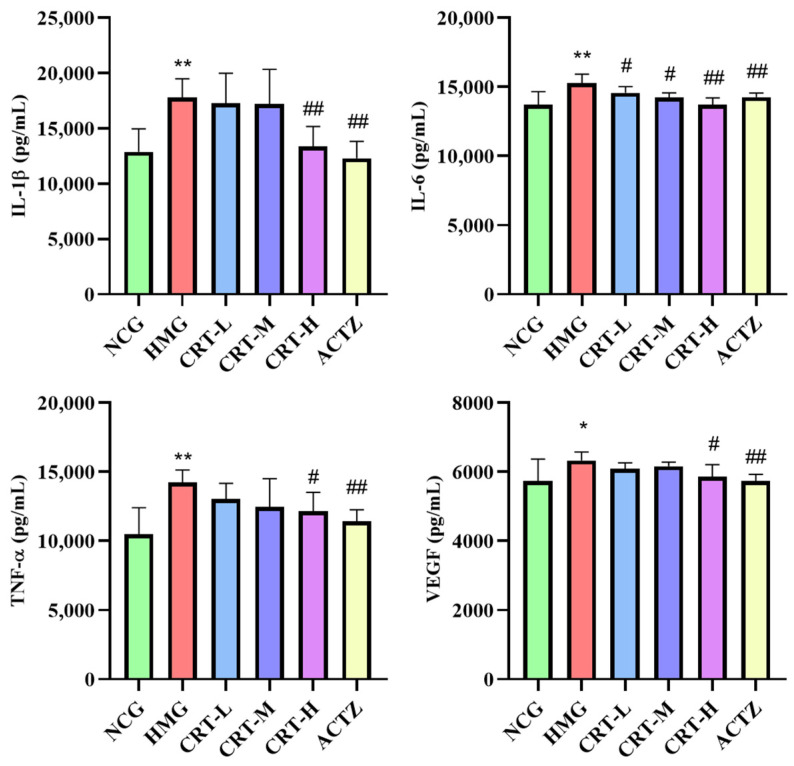
Crocetin reduces inflammatory factor in acute hypoxic injury in rats’ liver tissues. Data are expressed as mean ± SD (*n* = 8/group). Note: ** *p* < 0.01, * *p* < 0.05, NCG vs. HMG; ^##^ *p* < 0.01, ^#^ *p* < 0.05, HMG vs. medication groups; NCG: normal oxygen control group; HMG: hypoxia model group; CRT-L: crocetin low-dose group; CRT-M: crocetin medium-dose group; CRT-H: crocetin high-dose group; ACTZ: positive control group.

As shown in Figure 14, compared with NCG, the content of IL-1β (*p* < 0.01), IL-6 (*p* < 0.01), TNF-α (*p* < 0.01), and VEGF (*p* < 0.05) in the liver of HMG rats was significantly increased by 37.85%, 11.19%, 35.72%, and 10.43%.

After drug intervention, compared with HMG, the content of IL-1β in CRT-H and ACTZ groups decreased by 24.79% and 30.79%, respectively, and there were significant differences (*p* < 0.01). The content of IL-6 in CRT-L, CRT-M, CRT-H, and ACTZ groups decreased by4.45, 6.73%, 9.97%, and 6.52%, respectively (*p* < 0.05). The content of TNF-α in CRT-H and ACTZ groups decreased by 14.70% and 19.67%, respectively. There were significant differences (*p* < 0.05 or *p* < 0.01). The content of VEGF in CRT-H and ACTZ groups decreased by 7.51% and 9.28%, respectively. There were significant differences (*p* < 0.05).

##### Effect of Crocetin on Inflammatory Factors in Kidney Tissue of Hypoxic Rats

**Figure 15:**
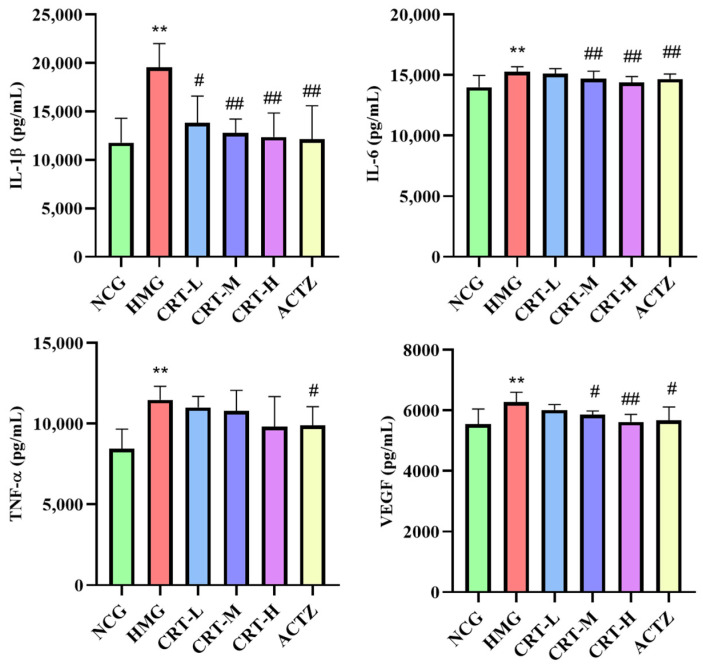
Crocetin reduces inflammatory factor in acute hypoxic injury in rats’ kidney tissues. Data are expressed as mean ± SD (*n* = 8/group). Note: ** *p* < 0.01, NCG vs. HMG; ^##^ *p* < 0.01, ^#^ *p* < 0.05, HMG vs. medication groups; NCG: normal oxygen control group; HMG: hypoxia model group; CRT-L: crocetin low-dose group; CRT-M: crocetin medium-dose group; CRT-H: crocetin high-dose group; ACTZ: positive control group.

As shown in Figure 15, compared with NCG, the content of IL-1β (*p* < 0.01), IL-6 (*p* < 0.01), TNF-α (*p* < 0.01), and VEGF (*p* < 0.05) in the kidney of HMG rats was significantly increased by 66.17%, 9.10%, 35.95%, and 12.83%.

After drug intervention, compared with HMG, the content of IL-1β in CRT-H, and ACTZ groups decreased by 36.85% and 37.95%, respectively. There were significant differences (*p* < 0.01). The content of IL-6 in CRT-H and ACTZ groups decreased by 6.03% and 3.97%, respectively. There were significant differences (*p* < 0.05). The content of TNF-α in ACTZ groups decreased by 13.68%, (*p* < 0.05). The content of VEGF in CRT-M, CRT-H, and ACTZ groups decreased by 6.57%, 10.33%, and 9.54%, respectively. There were significant differences (*p* < 0.05 or *p* < 0.01).

## 3. Discussion

Crocetin is the main active ingredient of *Crocus sativus* L. and mainly exists in the stigma of its plants. Crocetin is a polyunsaturated conjugated enoic acid structure with seven conjugated double bonds, which can form glycosidic bonds with gentian disaccharides or glucose to generate different crocin. Its solubility is poor in water and most organic solvents (except pyridine and dimethyl sulfoxide) [15]. According to reports, the fruit of gardenia also contains crocetin [16]. In this study, we prepared crocetin from the fruit of *Gardenia jasminoides* Ellis (content and yield were tested as 96.18% and 1.36%, respectively). In the reducing ability and free radical scavenging ability test, crocetin was found with strong scavenging ability for •O_2_^−^, H_2_O_2_, and •OH^−^, and strong antioxidant activity. In vitro anti-hypoxia tests with PC_12_ cell, normobaric hypoxia, and sodium nitrite hypoxia tests with mice proved crocetin had a strong anti-hypoxia effect.

**Figure 16:**
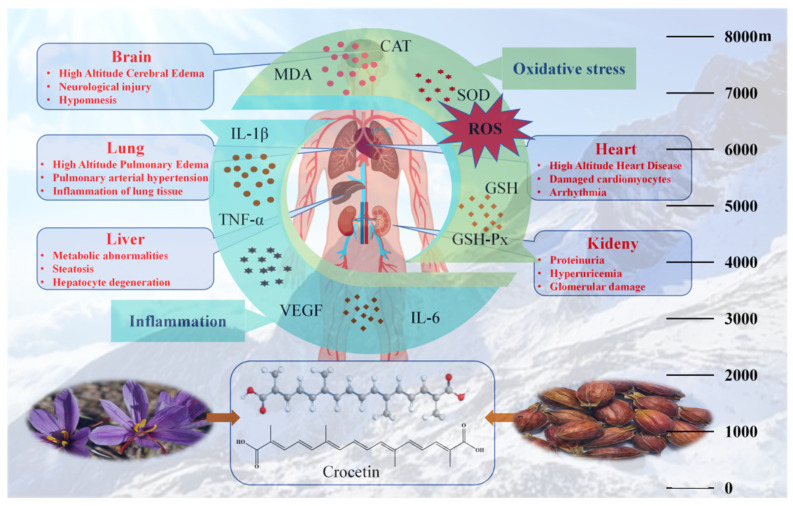
The damage of high-altitude hypoxia to vital organs and the protective effect of crocetin on various tissues. High altitude hypoxia can lead to oxidative stress and inflammation outbreaks, which in turn cause damage to the brain, lungs, heart, liver, and kidney organs, manifested as corresponding high-altitude hypoxic diseases in various tissues. Crocetin exists in the stigma of saffron and the fruit of traditional Chinese medicine *Gardenia jasminoides* and has a good inhibitory effect on oxidative stress and inflammation of various organs and tissues caused by high-altitude hypoxia.

In this experiment, the acute hypoxic injury rat model was established by simulating the plateau environment at an altitude of 8000 m. The water content of the brain and lung, the pathological changes, and the levels of oxidative stress indexes and inflammatory factors in the brain, lung, heart, liver, and kidney were tested to prove the protective effect of crocetin on high-altitude hypoxia rats. The results showed that the oxidative stress indicators and inflammatory factors in important tissues and organs of the HM group rats were significantly increased, indicating that high-altitude hypoxia can lead to systemic oxidative stress and inflammation outbreaks and cause pathological damage to the brain, lungs, liver, and kidney tissues. Due to the different tolerances of different tissues to high altitude hypoxia, the degree of pathological damage in each tissue also varies. Among them, brain and lung damage is more obvious, while heart tissue is less obvious. The intervention of crocetin could ameliorate the pathological damage in brain, lung, liver, and kidney, reduce the water content of the brain and lung, reduce the content of MDA, and H_2_O_2_, increase the content of GSH, increase the activity of GSH-Px, and reduce IL-6, IL-1β, TNF-α, and VEGF levels in acute hypoxic injury rats. It was suggested that crocetin could relieve oxidative stress and inflammatory response to alleviate hypoxic impairment on vital organs in high-altitude hypoxia rats (Figure 16).

In the past decade, crocetin has been reported with potential antioxidant and anti-inflammatory activity [17,18]. Peng et al. found that crocetin pretreatment can effectively protect the uterus from I/R injury and inhibit oxidative stress. The mechanism may be related to the activation of Nrf2/HO-1 signaling pathway [19]. The effects of crocetin on oxidative stress include reducing MDA and NO levels, increasing GSH content, and increasing the activity of antioxidant enzymes (SOD, CAT, GPx).

More and more studies have proved that oxidative stress and inflammation played essential roles in diseases that are caused by high-altitude hypoxia [12,20,21]. ROS refers to the general term for peroxides that are related to oxygen metabolism in living organisms, including oxygen-containing free radicals and those that are prone to forming free radicals, including peroxides (H_2_O_2_), superoxides (O_2_^−^), hydroxyl radicals (OH^−^), etc. Oxidative phosphorylation is optimal at physiological oxygen concentrations, and any change in the direction of oxygen availability may lead to an increase in ROS production [3].

Upon reaching high altitude, the hypoxic environment causes an imbalance between oxidation and antioxidation, leading to an overproduction of free radicals and intensifying oxidative stress. As our experimental results reveal, following hypoxia, the levels of antioxidants like SOD, CAT, GSH, and GSH-Px in rats’ tissues decreased, whereas the levels of oxidative stress markers MDA and H_2_O_2_ increased. The current study is in agreement with the research of Xiong Y, et al. [22], who observed that hypoxia causes an increase in serum and liver pro-inflammatory cytokine release, liver ROS production, and MDA content in rats, as well as a decrease in liver SOD, CAT, and GSH-Px activity. The copious free radicals from oxidative stress attack biomolecules such as cell membranes, proteins, and nucleic acids, causing oxidative damage and triggering cell apoptosis and inflammatory responses, which ultimately worsen tissue damage and the incidence of high-altitude illnesses.

Furthermore, hypoxia can also lead to the outbreak of inflammation, and it has been reported that the contents of inflammatory factors TNF-α, IL-6, and IL-1β in the brain tissue of hypoxia rats were significantly increased [23]. A similar study showed that the levels of proinflammatory factors iNOS, IL-6, and IL-1β increased for 30 consecutive days in Wistar rats at an altitude of 3658 m [24].

Abnormal expression of inflammatory factors (TNF-α, IL-1, IL-6) and chemokines (IL-8, CXC-4), induction of cyclooxygenase-2 (COX-2) and inducible nitric oxide synthase (iNOS), and changes in microRNA expression play a key role in oxidative stress-induced inflammation [25]. VEGF is a vascular endothelial growth factor that can promote vascular permeability, vascular endothelial cell migration, proliferation, and angiogenesis. Studies have shown that the VEGF promoter contains HRE, which can bind to the transcription factor-HIF-1α, and hypoxia exposure can promote cell VEGF expression [26,27]. Our experimental results showed that after hypoxia, the levels of TNF-α, IL-1 β, IL-6, and VEGF in various tissues of rats significantly increased, indicating that there were inflammatory reactions in all tissues of rats after hypoxia.

Crocetin, a carotenoid, possesses potent antioxidant properties, confirmed by our free radical scavenging experiments. Following intraperitoneal injections in simulated high-altitude hypoxia rats, crocetin notably reduced oxidative stress and inflammatory factors in the brain, lungs, heart, liver, and kidney tissues, showcasing its effective antioxidant and anti-inflammatory properties. It presents as a promising potential treatment for high-altitude hypoxia. Despite crocetin’s excellent anti-hypoxia activity, its specific polyunsaturated conjugated enoic acid structure contributes to its instability, insolubility, and low bioavailability, thereby limiting its application potential. The solubility and oral bioavailability issues have also influenced the administration method of crocetin. In this study, we used intraperitoneal injections. Future research could focus on enhancing the dosage form, such as integrating crocetin with nano-delivery systems, to enhance its solubility, stability, and bioavailability, thus better leveraging its anti-hypoxia properties. Modifying the dosage form can also alter the administration method of crocetin, rendering it safer and more convenient for the prevention and treatment of high-altitude hypoxia injuries.

## 4. Materials and Methods

### 4.1. Materials and Reagents

The fruit of *Gardenia jasminoides* Ellis was purchased from the medicinal materials market of An-Guo and was authenticated by professor Jinhui Wang of the Department of Pharmacy, Affiliated Hospital of Gansu University of Chinese Medicine, Lanzhou, China.

Polyamide (30 mesh) was purchased from Huangyan Resin Chemical Co., Ltd. (Taizhou, China). All other chemicals were of analytical grade or purchased from Rionlon Bohua (Tianjin, China) Pharmaceutical & Chemical Co., Ltd. The superoxide dismutase (SOD, No:A001-1-2), glutathione peroxidase (GSH-Px, No:A005-1-2), hydrogen peroxide (H_2_O_2_, No:E004-1-1), and catalase (CAT, No:A007-1-1) kits were purchased from Biyuntian Biotechnology Co., Ltd. (Shanghai, China).

The malondialdehyde (MDA, No:A003-1-1) and glutathione (GSH, No:A006-1-1) kits were purchased from Jiancheng Bioengineering Research Institute (Nanjing, China). IL-1β (No:SEKR-0002), IL-6 (No:SEKR-0005), VEGF (No:SEKR-0032), and TNF-α (No:SEKR-0009) ELISA kits for rats were purchased from Xinbosheng Biotechnology Co., Ltd. (Shenzheng, China). The TaKaRa MiniBEST Universal RNA Extraction Kit, PrimeScript™ RT Master Mix, TB Green™ Premix EX Taq™ II, and primers were bought from TaKaRa Biotechnology (Dalian, China).

### 4.2. Equipment

DYC-9070 simulated plateau hypobaric and hypoxic animal experimental cabin (Fenglei Aviation Ordnance Co., Ltd., Anshun, China); 3K15 High-Speed Refrigerated Centrifuge (Sigma, Livonia, MI, USA); SpectraMax^®^ i3 Automatic Fluorescence Microplate Reader (Molecular Devices, San Jose, CA, USA); BP210S Electronic Balance (Sartorius, Göttingen, Germany); DK-8A Electric Constant Temperature Bath (Jinghong Experimental Equipment Co., Ltd., Shanghai, China); E200 Optical Microscope (Nikon, Tokyo, Japan); Electric Homogenizer (Polytron@PT 1200E, Kinematica AG, Malters, Switzerland); Freeze dryer (Telster LyoQuest-55 plus type in Spain).

### 4.3. Prepared Crocetin from the Fruit of Gardenia Jasminoides Ellis and Its Content Determination

An amount of 500 g of powder of the fruit of *Gardenia jasminoides* Ellis was extracted at 100 °C with 5000 mL water three times. The aqueous extract was cooled, filtered, and flowed through polyamide chromatographic column (500 g, Φ 8 cm × 35 cm), which had been depurated with 95% alcohol and distilled water. The polyamide column was eluted with distilled water until the Molish reaction of elution was negative. Next, 1000 mL of 90% alcohol was used to elute the polyamide column, and the gardenia yellow pigment was obtained after the alcohol elution was vacuum-dried at 60 °C. An amount of 3 mol/L NaOH solution was added into the gardenia yellow pigment with a material-to-liquid ratio of 1:6. This reacted at a constant temperature of 55 °C for 60 min, then was cooled to room temperature and filtered with suction. The filter cake was washed with purified water 2~3 times and then dissolved in an appropriate amount of purified water, then filtered with the microporous membrane; 20% hydrochloric acid was add to the filtrate to adjust the pH to 2–3, and this was filtered to obtain crocetin.

The chromatographic conditions were determined: the chromatographic column was a Waters Symmetry C18 liquid chromatographic column (4.6 mm × 150 mm, 5 μm); the gradient elution was performed with acetonitrile (A)–0.6% formic acid aqueous solution (B) as the mobile phase, and the elution procedure was as follows: 0~12 min, 10%→70% A; 12~16 min, 70%→85% A; 16~25 min, 85% A; 25–26 min, 85%→100% A; 26–29 min, 100% A; 29–32 min, 100%→10% A; 32–35 min, 10% A. The flow rate was 1.0 mL/min; the detection wavelength was 423 nm; the column temperature was 37.0 °C; the injection volume was 20 μL; and the analysis time was 35 min.

**Figure 17:**
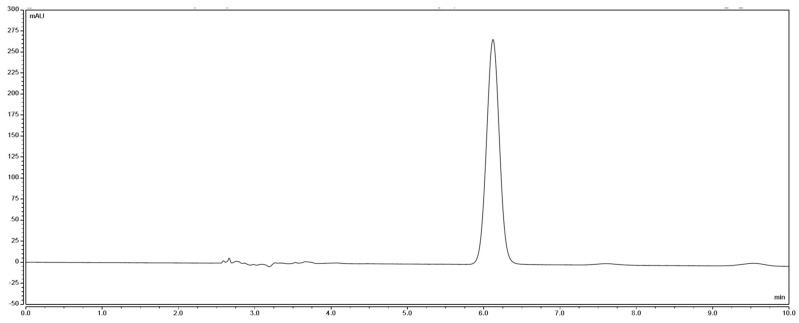
High-performance liquid chromatogram of the crocetin standard.

**Figure 18:**
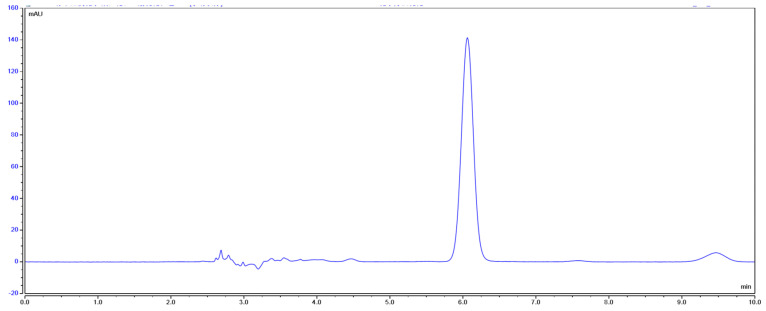
High-performance liquid chromatogram of the crocetin samples.

According to the high-performance liquid chromatography (Figure 17 and Figure 18), it can be seen that the prepared crocetin sample and crocetin standard have the same retention time under the same chromatographic conditions. And the manual integration result shows that the relative peak area of the sample is 96.18%, indicating that the purity of the sample is greater than 95%.

### 4.4. Reducing Ability and Radical Scavenging Ability Test

An amount of 10.00 mg crocetin was dissolved with 0.5 mL DMSO and diluted with distilled water in a 50 mL volumetric flask to obtain 200.00 μg/mL crocetin solution. This solution was further diluted with distilled water to 1.00, 2.50, 5.00, 10.00, 25.00, 50.00, 100.00, and 200.00 μg/mL series solution, respectively. The series solution of ascorbic acid was prepared with the same method.

The in vitro reducing ability of crocetin was determined by the Prussian blue reaction. The luminol dimethyl sulfoxide sodium hydroxide luminescence system was used to determine the superoxide anion scavenging capacity of crocetin, the luminol H_2_O_2_ chemiluminescence system was used to determine the H_2_O_2_ scavenging capacity of crocetin, and the luminol FeSO_4_-H_2_O_2_ chemiluminescence system was used to determine the OH^−^ scavenging capacity of crocetin. The inhibition rate and half-inhibitory concentration (IC_50_) were calculated to evaluate the reducing ability and scavenging ability of •O_2_^−^, H_2_O_2_, and •OH^−^.

### 4.5. In Vitro Anti-Hypoxia Test with PC_12_ Cell

The PC_12_ cell experiment was divided into a normal control group (NC), a hypobaric hypoxia group (HH), and a low dose of crocetin group (CRT-L, 25 μMol/L), medium dose of crocetin group (CRT-M, 50 μMol/L), and high dose of crocetin group (CRT-H, 100 μMol/L). Among them, crocetin was soluted with DMSO and added in medium for crocetin groups. The NC group was cultured in a normoxia constant temperature incubator for 36 h. The other groups were cultured in a hypoxic constant temperature incubator (1% O_2_, 5% CO_2_, and 37℃) for 36 h. Then, the culture medium was discarded, and 100 μL 100% CCK-8 medium was added and cultured for another 2 h. The absorbance at 450 nm was measured using an enzyme-linked immunosorbent assay (ELISA) to calculate cell viability.

### 4.6. Animals

SPF male BALB/C mice (aged 4–5 weeks with an average body weight of 20 ± 2 g) and Wistar rats (aged 6–8 weeks with an average body weight of 200 ± 20 g) were purchased from the Beijing Vital River Laboratory Animal Technology Co., Ltd. (Beijing, China, License No. SCXK (Jing) 2021-0011). The animals were fed in the rearing room with a temperature of 20–25 °C and a relative humidity of 40–70%. Alternating light and darkness every 12 h in the rearing room, the rats freely fed and drank water. The adaptation period was 1 week before the start of the investigation.

The experimental protocol used in this study was approved by the 940th Hospital of Joint Logistic Support Force of Chinese People’s Liberation Army, with approval number: 2022KYLL031.

### 4.7. Normobaric Hypoxia and Sodium Nitrite Hypoxia Tests

A total of 50 BALB/c mice were randomly divided into the control group (sterile water, 10 mL/Kg), positive control group (acetazolamide, 100 mg/kg/d), crocetin low-dose group (15 mg/kg/d), crocetin medium-dose group (30 mg/kg/d), and crocetin high-dose group (60 mg/kg/d). The drugs were given twice, at 12 h and 30 min before normobaric hypoxia. All mice were placed in a 200 mL jar with 5 g of soda lime (absorption of carbon dioxide and water). The bottles were covered tightly with Vaseline, and time was counted immediately. The mice were observed, and the time when the mice died due to lack of oxygen was recorded.

The animals were divided into groups and administered as described in the normobaric hypoxia test. One hour after the last administration, 300 mg/kg sodium nitrite solution was injected into the abdominal cavity. The mice were observed, and the time when the mice died due to lack of oxygen was recorded.

### 4.8. Establishment of High Altitude Hypoxia Rat Model and Administration

The 72 experimental rats were randomly divided into the normoxic control group (NCG, 0.5% CMC-Na, 10 mL/kg), hypoxia model group (HMG, 0.5% CMC-Na, 10 mL/kg), crocetin-low dose group (CRT-L,10 mg/kg), crocetin-medium dose group (CRT-M, 20 mg/kg), crocetin-high dose group (CRT-H, 40 mg/kg), and acetazolamide group (ACTZ, 70 mg/kg). Under the condition of an SPF environment, the rats began the experiment after 3 days of adaptive feeding.

The DYC-9070 simulated altitude hypoxia animal experimental cabin used in the experiment consists of three parts: control system, experimental cabin, and simulation cabin. The experimental cabin was the one where the rats were dissected after the hypoxia model was established, and the simulated altitude is 4000 m; the simulation cabin is a place where experimental animals were exposed to hypoxia and could simulate altitudes above 8000 m; the control system controlled them. There were independent valves between the experimental cabin and the simulation cabin, as well as between the experimental cabin and the external environment, that is, internal valves and external valves.

The specific administration methods of the six groups of rats were as follows: the rats in the normoxic control group (NCG) and the hypoxia model group (HMG) were intraperitoneally injected with 0.5% CMC-Na, and the other four groups were intraperitoneally injected with the corresponding doses, respectively, 30 min before hypoxia, 12 h after hypoxia, and 24 h after hypoxia. A total of three times were administered, and the hypoxia exposure time was 24 h. During this period, the rats were fed and drank freely. Within 24 h of hypoxia, the on-duty personnel were arranged to observe the food intake and active state of the rats by monitoring.

**Figure 19:**
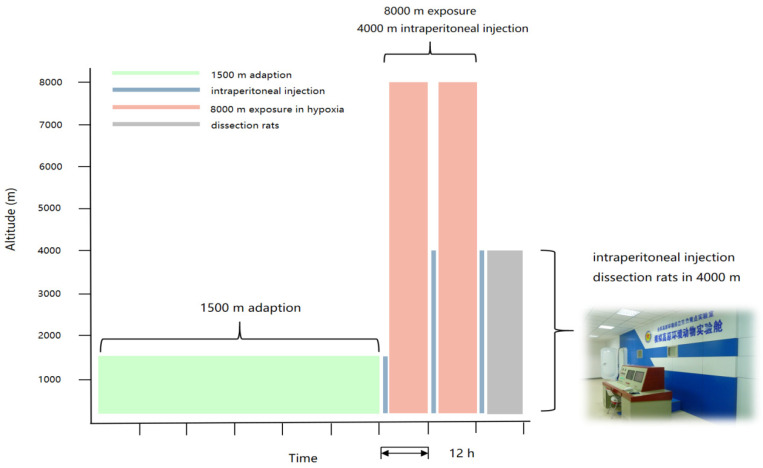
Establishment of high-altitude hypoxia rat model and administration.

The normoxia control group (NCG) was raised in the experimental animal department during the experiment, and the other five groups of rats were placed in a simulated cabin at an altitude of 8000 m after the first administration. After 12 h of hypoxia in the experimental rats, the experimenters entered the experimental cabin. The extravehicular experimenters used the control system to rise at a constant speed of 4 m/s to the altitude of 4000 m in the experimental cabin, and at the same time, they dropped at a constant speed of 20 m/s to the altitude of 4000 m in the simulation cabin. After the two cabins became stable, the experimenters could open the inner valve and enter the simulation cabin for the second intraperitoneal injection. After 24 h of hypoxia in the experimental rats, the experimenters entered the cabin for the third administration. After 30 min of administration, the rats were decapitated and killed to take tissues. At the same time, the extravehicular experimenters handled NCG rats in the same way (Figure 19).

### 4.9. Determination of Brain and Lung Water Content

The 1/2 tissue of the upper part of the left brain and the upper lobe of the left lung were placed on the weighing paper to weigh the wet weight. After weighing, they were placed in a constant temperature blast oven at 60 °C for more than 72 h until the difference between the two weighing dry weights did not exceed 0.002 g. The water content of rat brain and lung tissue was calculated according to Formula (1):

(1) $$Water\;content\;(\%)=\frac{wet\;weight-dry\;weight}{wet\;weight}\times 100\%$$

### 4.10. HE Staining

After the high-altitude hypoxia model was established, rats were decapitated, and the right half brain was fixed in 4% paraformaldehyde solution, embedded, and sectioned for HE staining. The histopathologic changes were observed under the microscope (×400).

### 4.11. Biochemical Analysis and Enzyme-Linked Immunosorbent Assay (ELISA)

An amount of 180–220 mg tissue was weighed to make 10% homogenate with cold 1×PBS. The supernatant was taken after centrifugation (10,000 r/min, 10 min, 4 °C). Followed the kit instructions, MDA, H_2_O_2_, GSH, GSH-Px, SOD, CAT, IL-1β, IL-6, TNF-α, and VEGF were determined.

### 4.12. Statistical Analysis

Statistical analysis was performed using SPSS 26.0 software. The data were expressed as the mean ± standard deviation (SD) ( $\bar{x}$ ± SD), and one-way analysis of variance (ANOVA) was used for further pairwise comparison using the LSD-*t* test. *p* < 0.05 was considered a significant difference.

## 5. Conclusions

In summary, the current findings demonstrate that crocetin exhibits good anti-hypoxia effects in both in vitro and in vivo models. In this regard, crocetin has strong free radical scavenging ability and can enhance the vitality of PC_12_ cells under hypoxic conditions and prolong the survival time of mice with different hypoxia models. More importantly, crocetin restored the oxidative and antioxidant balance in important organs of simulated high-altitude hypoxia rats, reducing oxidative stress and inflammatory reactions, thereby protecting the important organs of simulated high-altitude hypoxia rats from damage caused by hypoxia. Therefore, we recommend using crocetin as a protective agent to combat tissue damage caused by high-altitude hypoxia. The prevention and treatment of high-altitude diseases are based on our understanding of the physiological and pathological changes in people and bodies in high-altitude areas. Further research on the pathophysiological mechanisms of high-altitude diseases can improve our understanding of their precise therapeutic effects. However, crocetin has not yet been applied clinically to treat altitude illnesses due to its unique structure. Therefore, while studying the anti-hypoxic effects of crocetin, attention should also be paid to the drug’s administration method, dosage form, and safety. It is of great significance for it to enter clinical research as soon as possible.

## Figures and Tables

**Table 1:** Half concentration of crocetin for each radical group IC50 (*n* = 3).

Group	IC_50_ (μg/mL)		
	•O_2_^−^	H_2_O_2_	•OH^−^
ascorbic acid	89.29 ± 1.26	23.71 ± 0.10	14.82 ± 0.05
Crocetin	6.39 ± 0.15 *	4.67 ± 0.12 *	8.63 ± 0.05

Note: Compared with the ascorbate control group, * *p* < 0.001.

## Data Availability

The data used to support the findings of this study are available from the corresponding author upon request.
