# Correction: Yang et al. Evaluation of Crocetin as a Protective Agent in High Altitude Hypoxia-Induced Organ Damage. *Pharmaceuticals* 2024, *17*, 985

**DOI:** 10.3390/ph18111716

**Published:** 2025-11-12

**Authors:** Jun Yang, Kai Luo, Ziliang Guo, Renjie Wang, Qingyuan Qian, Shuhe Ma, Maoxing Li, Yue Gao

**Affiliations:** 1 College of Pharmacy, Gansu University of Chinese Medicine, Lanzhou 730000, China; yangjunzy@163.com (J.Y.); 18294623280@163.com (K.L.); wangrenjiegy@163.com (R.W.); mclsxka@163.com (S.M.); 2 Department of Pharmaceutical Sciences, Beijing Institute of Radiation Medicine, Beijing 100850, China; q15888906749@163.com; 3 College of Pharmacy, Lanzhou University, Lanzhou 730000, China; guozl20@lzu.edu.cn; 4 National Key Laboratory of Kidney Diseases, Beijing 100850, China

## Error in Figure

In the original publication [1], there was a mistake in Figure 5 as published. The panels C and D of the heart HE staining in Figure 5 were duplicated due to a copy–paste error. The corrected Figure 5 appears below.

The authors state that the scientific conclusions are unaffected. This correction was approved by the academic editor. The original publication has also been updated.

## Figures and Tables

**Figure 5:**
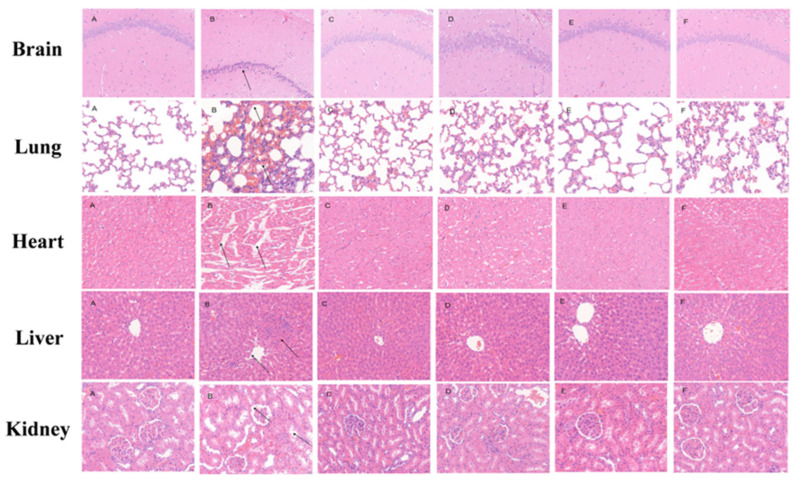
Histopathological changes of vital organs in high-altitude hypoxia rats (×400). (**A**) Normoxic control group (NCG), (**B**) hypoxia model group (HMG), (**C**) crocetin low-dose group (CRT-L, 10 mg/kg), (**D**) crocetin medium-dose group (CRT-M, 20 mg/kg), (**E**) crocetin high-dose group (CRT-H, 40 mg/kg), (**F**) acetazolamide group (ACTZ, 70 mg/kg).
